# Pleural Fluid Biomarkers of Pediatric Parapneumonic Effusion

**DOI:** 10.3390/diagnostics15091086

**Published:** 2025-04-24

**Authors:** Jose D. Santotoribio, David Nuñez-Jurado, Jose L. Rubio-Prieto, Juan M. Guerrero, Juan Corral-Pérez, Juan J. Fernández-Alba

**Affiliations:** 1 Department of Laboratory Medicine, Puerto Real University Hospital, 11510 Cadiz, Spain; 2 Instituto de Investigación e Innovación Biomédica de Cádiz (INiBICA), 11009 Cadiz, Spain; 3 ExPhy Research Group, Department of Physical Education, Cadiz University, 11519 Cadiz, Spain; 4 Department of Clinical Biochemistry, Virgen del Rocío University Hospital, 41013 Seville, Spain; david.nunez.jurado.sspa@juntadeandalucia.es (D.N.-J.); jose.rubio.prieto.sspa@juntadeandalucia.es (J.L.R.-P.);; 5 Department of Molecular Biology, Biochemistry and Immunology, Seville University School of Medicine, 41009 Seville, Spain; 6 Neuro-Inmuno-Endocrinología Molecular Group, Instituto de Biomedicina de Sevilla (IBIS), 41013 Seville, Spain; 7 Department of Obstetrics and Gynecology, Hospital Universitario de Puerto Real, 11510 Puerto Real, Spain

**Keywords:** pleural fluid, childhood, biochemical parameter

## Abstract

**Background/Objectives:** Parapneumonic pleural effusion (PPE) secondary to community-acquired pneumonia is the most common cause of pediatric pleural effusion. This study aimed to evaluate the pleural fluid characteristics of pediatric patients with PPE and to compare biomarkers between infants (1–12 months) and children (1–14 years). **Methods:** Fifty-four pediatric patients (14 infants and 40 children) with PPE were included. Pleural fluid samples were analyzed for white blood cell (WBC) count, glucose, total protein, lactate dehydrogenase (LDH), adenosine deaminase (ADA), and pH levels. Differences between age groups and correlations between age and pleural fluid biomarkers were assessed. **Results:** Most pediatric PPE cases exhibited biochemical characteristics consistent with pleural exudate: WBC > 1000 cells/µL, total protein > 3 g/dL, LDH > 200 U/L. Infants showed a predominance of mononuclear WBC, while children exhibited a predominance of polymorphonuclear WBC. Glucose levels were higher, and total protein levels were lower in infants compared to children. Age was positively correlated with polymorphonuclear WBC percentage (rho = 0.509, *p* < 0.001) and protein levels (rho = 0.622, *p* < 0.001), whereas glucose levels were negatively correlated with age (rho = −0.274, *p* = 0.043). **Conclusions:** Age-specific differences in pleural fluid biomarkers were observed in pediatric patients with PPE, suggesting a more robust and acute inflammatory response in children compared to infants. These findings underscore the importance of considering age-related variations in the inflammatory response when diagnosing and managing PPE in pediatric populations.

## 1. Introduction

Pediatric pleural effusion remains a significant clinical concern that requires regular evaluation by pediatric physicians despite advancements in diagnostic and therapeutic approaches. Pleural effusion can result from various causes in the pediatric population, with parapneumonic pleural effusion (PPE) secondary to community-acquired pneumonia being particularly common. Notably, approximately 10% to 30% of pneumonia cases in children may evolve into PPE, emphasizing the need for vigilant monitoring of respiratory infections in this demographic [1].

Among the pathogens associated with PPE, Streptococcus pneumoniae is the most common causative agent. This bacterium is well known for causing pneumonia and its complications, including pleural effusion, in pediatric patients. Recognizing Streptococcus pneumoniae as a leading culprit underscores the importance of early detection, prompt diagnosis, and appropriate management strategies to mitigate the risk of progression to PPE and its complications [2].

Timely evaluation and management of pleural effusion, especially when related to pulmonary infections, are crucial for preventing severe consequences in vulnerable pediatric populations. Although microbiological assessment of pleural fluid is highly specific for diagnosing PPE, it often lacks sensitivity and requires prolonged culture times [3]. Therefore, cytochemical analysis of pleural fluid remains essential for a comprehensive diagnosis. This analysis evaluates cellular components, such as white blood cells, and biomarkers of exudative effusions—including total proteins and lactate dehydrogenase (LDH)—as well as other parameters like glucose consumption, adenosine deaminase (ADA), and pH. Together, these measurements provide valuable insights into the inflammatory response, cellular composition, and underlying pathophysiology of pleural effusions.

Cytochemical analysis is pivotal in distinguishing infectious from non-infectious causes of pleural effusion, thereby guiding appropriate treatment strategies and improving patient outcomes. Traditionally, in cases of PPE, pleural fluid typically exhibits an exudative pattern with white blood cell (WBC) counts exceeding 1000 cells/µL [4], a predominance of polymorphonuclear cells [5], total protein levels surpassing 3 g/dL [6], and LDH levels above 200 U/L [7]. Additionally, features of infectious pleuritis often include reduced glucose levels (<60 mg/dL) [6], elevated ADA levels (>35 U/L) [8,9], and acidic pH (<7.35) [10,11].

It is important to recognize that PPE can evolve. Initially, a simple PPE may progress to a complicated PPE. While a simple PPE generally responds well to antibiotics, a complicated PPE often requires interventions such as thoracic drainage. PPE represents a spectrum of disease, ranging from simple effusions to complicated effusions, with empyema representing the most severe form, characterized by the presence of frank pus in the pleural space [3]. This study aims to evaluate the pleural fluid characteristics of pediatric patients with PPE, including the full spectrum of disease progression to empyema. By integrating microbiological and cytochemical findings, clinicians can better understand the etiology and severity of pleural effusion, distinguish between simple and complicated PPE, and tailor treatment approaches accordingly. Notably, patients with complicated PPE often present with lower pH and glucose levels and higher LDH activity, reflecting the increased metabolic activity of inflammatory cells and bacteria in the pleural cavity [12]. Pleural fluid pH has demonstrated high diagnostic accuracy for identifying complicated PPE, with drainage recommended when pH falls below 7.2, or when glucose is below 40 mg/dL, LDH exceeds 1000 U/L, or pleural fluid culture is positive [10,11].

Despite the existence of several studies on pediatric pleural effusions [13,14,15], a detailed analysis of pleural fluid biomarkers remains scarce, with only a limited number of articles addressing this issue [16]. Moreover, these studies have not differentiated between age groups, despite recommendations by other authors [17]. Conducting such comparative studies can help identify age-specific risk factors, clinical patterns, and prognostic indicators associated with PPE. Therefore, this study aims to evaluate the pleural fluid characteristics of pediatric patients with PPE and to compare pleural fluid biomarkers between infants (1–12 months) and children (1–14 years).

## 2. Materials and Methods

The multicenter cross-sectional study was conducted in southern Spain, specifically in Andalusia, and involved two prominent medical centers: Puerto Real University Hospital in Cádiz (PRUH) and Virgen del Rocío University Hospital in Seville (VRUH). These hospitals were selected for their expertise, resources, and accessibility, ensuring a robust and representative sample for this study.

The research strictly adhered to the ethical guidelines outlined in the World Medical Association Declaration of Helsinki [18]. Furthermore, the study protocol and procedures received approval from the Institutional Research Ethics Committee of both participating hospitals. This approval underscored the ethical integrity and scientific rigor of this study, affirming its compliance with established ethical standards and ensuring the protection of participants’ rights and welfare.

### 2.1. Patients

This study was conducted between September 2021 and May 2023 at two medical centers concurrently. Before any medical procedures, the legal guardians of the patients received a comprehensive informed consent form detailing this study’s nature, objectives, potential risks, and benefits. After a thorough review, the guardians signed the consent form, permitting their child’s participation in the research.

Eligibility criteria focused on patients with pleural effusion requiring diagnostic thoracentesis. Participants were aged from 1 month to 14 years, regardless of gender. Specifically, inclusion criteria encompassed cases of parapneumonic pleural effusion (PPE), characterized by indicators such as purulent pleural fluid, positive culture results for pathogens other than Mycobacterium tuberculosis, or pleural effusions associated with bacterial pneumonia, lung abscesses, or bronchiectasis as well as empyema, defined by the presence of frank pus obtained via thoracentesis. Only pleural fluids obtained during the initial thoracentesis were considered for analysis; fluids from subsequent procedures were excluded.

To facilitate analysis and interpretation, patients were stratified into two age groups: infants (1–12 months) and children (1–14 years) [19]. This stratification enabled a more detailed exploration of age-related variations and trends in the presentation and characteristics of pediatric pleural effusions.

### 2.2. Data Collection

During the data collection phase, demographic information (age, sex, and relevant clinical details) was meticulously gathered via electronic chart review. Laboratory analyses of pleural fluid were performed to characterize its composition and identify potential markers of pathology, including ADA levels, glucose concentration, total protein content, LDH activity, and both total and differential white blood cell counts, as well as red blood cell counts. Additionally, all culture results from the pleural fluid samples were recorded. Cultures were evaluated using solid media (blood agar, chocolate agar, MacConkey agar, and anaerobic media such as KV and Schaedler) and enriched liquid media (thioglycollate broth), to ensure accurate identification of pathogens or microbial agents.

Pleural fluid samples were obtained from each patient via thoracentesis using plain tubes without anticoagulants and a gasometry syringe to ensure accuracy. At PRUH, cell counts were determined using the UF-4000 system (Sysmex España, Sant Just Desvern, Spain), while at VRUH, a similar analysis was performed with the XN-10 system (Sysmex España). Following collection, samples were centrifuged at 4000 rpm for 7 min to separate the supernatant for further analysis.

Simple parapneumonic effusion is defined by pleural fluid that lacks biochemical and/or microbiological signs of infection (slightly decreased pH and glucose, slightly increased LDH, and negative cultures). Complicated parapneumonic effusion is characterized by pleural fluid that shows micro-biochemical signs of infection (very low pH and glucose, very high LDH, and/or positive cultures). Empyema is defined by the presence of pus in the pleural fluid. Therefore, the primary diagnostic criterion for parapneumonic pleural effusion is its association with bacterial pneumonia, lung abscesses, or bronchiectasis (our main inclusion criterion).

Comprehensive biochemical analyses were subsequently conducted on all pleural fluid samples. At PRUH, these analyses were performed using the Alinity CI system (Abbott Laboratories, Abbott Park, IL, USA), whereas at VRUH, the Cobas 8000 system (Roche Diagnostics, Rotkreuz, Switzerland) was employed. Pleural fluid pH was measured separately using gasometry techniques: at PRUH with the GEM^®^ Premier 5000 system (Werfen, Barcelona, Spain) and at VRUH with the ABL90 FLEX system (Radiométrica Ibérica, Madrid, Spain).

### 2.3. Statistical Analysis

To assess the normality of our dataset, we applied the Kolmogorov–Smirnov test. Continuous variables not following a normal distribution are presented as median with interquartile range (IQR), while those with normal distribution are shown as mean ± standard deviation. Categorical variables are expressed as counts and percentages.

We used the chi-square test to explore associations between categorical variables across different age groups, focusing on typical pleural fluid characteristics in parapneumonic effusion (PPE). Pleural fluid biochemical typical characteristics of PPE include WBC > 1000 cells/µL [4], with a predominance of polymorphonuclear cells [5], total protein > 3 g/dL [6], LDH > 200 U/L [7], glucose < 60 mg/dL [7], ADA > 35 U/L [8,9], and pH < 7.35 [10,11].

For comparisons within each age group, we employed either the Mann–Whitney U test or the Independent Samples *t*-Test, depending on the normality of the variables. Spearman’s correlation was used for non-normally distributed variables, and Pearson’s correlation for normally distributed ones, to examine relationships between age and pleural fluid biomarkers. In addition to this, to account for the small sample size in our study, we performed a bootstrapped analysis of our data using 1000 imputations to confirm the significant differences and associations found in our statistical analysis.

All statistical analyses were performed using IBM SPSS Statistics version 26, with significance set at *p* < 0.05.

## 3. Results

This study enrolled 54 hospitalized patients diagnosed with PPE secondary to community-acquired pneumonia, comprising 20 males and 34 females. All patients were vaccinated according to the government vaccination schedule. Among these, 34 received treatment at VRUH and 20 at PRUH. Pleural fluid cultures were positive in 14 patients (25.5%): 13 cases identified Streptococcus pneumoniae and 1 case identified Klebsiella pneumoniae as the causative agent. The diagnostic classifications and treatments for all patients are detailed in Table 1.

The demographic data of the patients and variations in the levels of pleural fluid biomarkers are comprehensively outlined in Table 1. This analysis reveals intriguing differences across different age groups.

The pleural fluid of infants showed elevated glucose levels (*p* = 0.036) and lower concentration of proteins (*p* = 0.001) compared to children, and the majority of infants had exudated pleural effusions with mononuclear leukocyte predominance, potentially reflecting variations in metabolic and inflammatory processes in the pleural space.

**Figure 1:**
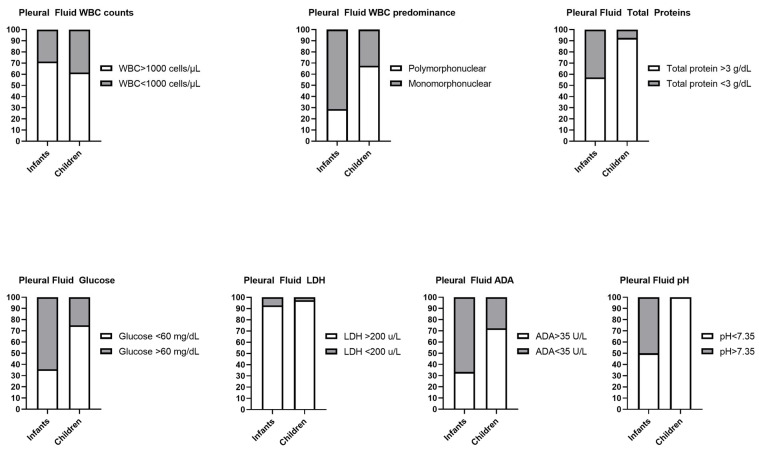
Distribution of patients with pleural fluid biochemical characteristics typical of PPE in infants and children. The bar figures show the percentage of patients (%). WBC: white blood cell; LDH: lactate dehydrogenase; ADA: adenosine deaminase.

The distribution of patients with pleural fluid biochemical characteristics typical of PPE in infants and children is visually depicted in Figure 1, providing a clear illustration of the differences observed between the two age groups.

The Spearman’s correlation coefficient revealed significant associations between age and pleural fluid biomarkers. Positive associations were observed between age and polymorphonuclear WBC percentage (rho = 0.485, *p* < 0.001), as well as protein levels (rho = 0.609, *p* < 0.001). Conversely, negative associations were found between age and glucose levels (rho = −0.262, *p* = 0.043). No further significant associations were identified, as depicted in Table 2.

## 4. Discussion

In this multicenter cross-sectional study of pediatric parapneumonic pleural effusion (PPE), we found differences in pleural fluid biomarker profiles between infants and older children. Specifically, infants showed a predominance of mononuclear white blood cells (WBCs), unlike their older counterparts. Additionally, significant variations were noted in total protein and glucose levels in the pleural fluid, with statistical correlations between these markers and age. These findings highlight important age-related differences in the biochemical profiles of PPE, which may reflect distinct underlying pathophysiological processes or responses to the condition in different age groups.

The pleural fluid cultures had low sensitivity, identifying pathogens in only about one-quarter of the samples. Streptococcus pneumoniae was the most common pathogen, consistent with findings from other studies [3]. The relatively low culture positivity rate observed in our study (25.5%) may be attributed to the frequent administration of antibiotics prior to thoracentesis, which can reduce bacterial viability and hinder microbiological growth. Additionally, although pleural fluid samples were cultured using solid and enriched liquid media, they were not inoculated into blood culture bottles, a technique known to enhance the recovery of pathogens in parapneumonic effusions.

The results of this study indicated that both age groups had WBC values exceeding 1000 cells/µL, consistent with the criteria for pleural exudates [4]. Typically, PPEs are characterized by an exudative nature with a predominance of polymorphonuclear WBCs (neutrophils) [5], which was observed in our pediatric sample. However, in infants, most PPE cases exhibited exudative characteristics, including elevated protein and LDH levels, but with a predominance of mononuclear WBCs, suggesting a predominantly lymphocytic inflammatory response. The neutrophil predominance in children compared to infants points to a more intense and acute inflammatory response in children, while infants showed a lymphocytic predominance.

PPE is characterized by total pleural fluid protein levels exceeding 3.0 g/dL, which was observed in both age groups [6]. However, a significant difference in protein levels was noted between infants and children, with children displaying higher levels compared to infants. Notably, a strong positive correlation (rho = 0.609) was found between total protein levels in pleural fluid and the age of the pediatric patients, indicating a marked increase in these biomarker levels as age increases. This indicates that, while age seems to significantly influence protein levels in pleural fluid (remaining significant even after bootstrapping analysis), the moderate association suggests the involvement of additional factors that should be investigated in future studies.

PPE typically leads to glucose consumption and increased lactate levels in pleural fluid, with glucose levels dropping below the 60 mg/dL threshold [6]. In our study, the children’s PPE cohort showed glucose levels consistently below this critical threshold, reflecting a similar metabolic pattern across age groups. Notably, the children subgroup had particularly low glucose values, with a median of 32.5 mg/dL. This difference was statistically significant when compared to the infant group, suggesting potential age-related variations in the metabolic response to pleural inflammation. However, the correlation between pleural fluid glucose levels and age was weak and inverse (rho = −0.262). This means that even though age appears to play an important role in glucose consumption in pleural fluid (and remained significant after the boot-strapping analysis), the weak association suggests that other factors contribute to this difference, which should be explored in future research.

Regarding pleural fluid LDH levels, PPE is typically characterized by LDH values exceeding 200 U/L, a threshold indicating exudates [7]. Remarkably, almost all participants in both age groups surpassed this threshold, and the majority exhibited high LDH levels (greater than 1000 U/L), exceeding the threshold for considering thoracic pleural drainage, highlighting a strong inflammatory response within the pleural cavity across the pediatric population studied. This emphasizes the importance of age-specific considerations when understanding the pathophysiology and severity of pediatric pleural effusions, with potential implications for clinical management strategies tailored to different age cohorts.

Regarding pleural fluid ADA levels, commonly used as a diagnostic tool for tuberculosis when values exceed 35–40 U/L, our study revealed that the majority of cases in the children’s group surpassed this threshold, despite the absence of tuberculosis. This observation underscores the limited specificity of ADA as a marker for tuberculosis, as elevated ADA levels can also occur in other conditions, such as parapneumonic pleural effusions and other forms of pleuritis [8,9]. In our region, even though tuberculosis prevalence is low, ADA is routinely measured in pleural fluid to provide additional information about the inflammatory process. The significant elevation of ADA levels in our study population highlights the need for caution when interpreting ADA results in pediatric pleural effusions, particularly in regions where tuberculosis is endemic. This finding underscores the importance of considering ADA results in the broader clinical context, integrating additional diagnostic modalities and patient factors to differentiate between tuberculosis and other pleural effusion etiologies. Further research is needed to identify more specific and sensitive biomarkers for tuberculosis and other pleural diseases in pediatric populations, improving diagnostic accuracy and patient care outcomes. No significant differences in ADA levels were found between the two pediatric groups.

Regarding pleural fluid pH levels, PPE is often associated with an acidic environment (pH < 7.35) [10]. Most cases in our study had pH values below this cut-off. The clinical relevance of pleural fluid pH is crucial, particularly in diagnosing complicated parapneumonic pleural effusion and determining the need for thoracic drainage tube treatment. Various studies have established different pleural fluid pH cut-off values for determining the need for thoracic drainage tube intervention. In adults, a pH below 7.2 typically indicates the need for drainage tube treatment [10]. For pediatric cases, some scientific societies recommend a lower pH cut-off of 7.0 to guide treatment decisions for thoracic drainage tube intervention [11]. Our study suggests the potential for using pleural fluid biomarkers to guide treatment decisions in pediatric PPE. However, due to the limitations of our sample size, we were unable to conduct a definitive analysis of the association between biomarker profiles and treatment outcomes. Larger studies are needed to confirm these preliminary observations and to develop evidence-based treatment algorithms.

Microbiological and cytochemical analyses of pleural fluid revealed that the majority of pediatric patients (85%) exhibited features consistent with complicated PPE. This included parameters such as pH < 7.0, glucose < 40 mg/dL, LDH > 1000 U/L, or positive pleural fluid cultures. These findings suggest heightened metabolic activity of inflammatory cells and bacteria within the pleural space in pediatric patients with PPE [10,11,12]. It is important to note that, unlike in adults, the decision to proceed with pleural drainage in children is not based exclusively on biochemical thresholds. Pediatric guidelines emphasize the importance of integrating clinical signs, radiological findings, and the patient’s overall condition when determining the need for intervention, rather than relying solely on pleural fluid parameters such as pH, glucose, or LDH.

An exudate with a predominance of PMNs in pleural fluid indicates a more robust and acute inflammatory response, whereas an exudate with a predominance of MNs is characteristic of chronic pleural processes. Consequently, patients with PPE almost always present an exudate with a predominance of PMNs in pleural fluid [5]. However, this pattern has not been observed in the majority of infant patients. The presence of exudates with a mononuclear predominance in most infant patients could be attributed to their higher proportion of naïve T cells, whereas older children have a greater number of memory T cells capable of mounting a stronger immune response [20]. Additionally, infants exhibit delayed maturation of immune responses, which affects their ability to mount effective long-term immunity [21], which could be associated with the more robust and acute inflammation found in our children’s population. Recognizing these developmental variations is essential for optimizing immunization strategies and tailoring therapeutic interventions to protect vulnerable populations.

This study has several limitations, the most notable being the relatively small sample size, which was drawn from specific regions in southern Spain and may restrict the generalizability of the findings. As a result, the observed trends might not apply to wider populations, highlighting the need for future research to validate these findings. In addition, these foresight studies may include a more significant number of pediatric patients with PPE, both treated and untreated with pleural drainage, which is needed to determine cut-off points for the decision to use pleural drainage in both infants and children, requiring a minimum of 37 participants from each patient group or subgroup, as suggested by previously proposed approaches [22]. One limitation of the current analysis is that pleural fluid was not routinely inoculated into blood culture bottles, a technique that may increase the yield of positive cultures, particularly in cases with low bacterial burden. Future studies should consider this approach to enhance pathogen detection. While we observed descriptive trends suggesting that patients receiving pleural drainage tended to have more deranged biomarker profiles (e.g., lower glucose and lower pH), we lacked the statistical power to determine the significance of these associations. Future studies with larger cohorts are needed to investigate this relationship and to identify potential biomarker-based predictors of treatment response. Furthermore, increasing the sample size will also enable the implementation of innovative risk prediction models using machine learning systems, as well as decision trees or random forests, as applied by other authors in recently published studies on other diseases [23]. One limitation of the current analysis is the lack of access to potential confounders, which prevented the inclusion of multivariate models to adjust for their influence. Future studies should aim to incorporate a broader range of covariates to provide a more comprehensive understanding of the independent effects of age on biomarker profiles, potentially using advanced multivariate techniques such as regression modeling or machine learning approaches. Another limitation of this study is the lack of blood sample parameters that could complement the results obtained in this study.

Despite this, this study provides valuable insights into the biochemical profiles of pediatric pleural effusions across different age groups. One of its strengths is the comprehensive data collection from two distinct hospitals, enhancing the robustness of the findings by incorporating diverse patient demographics and clinical practices. Additionally, the inclusion of various laboratory tests allows for a detailed understanding of the biochemical characteristics of pleural fluid in pediatric patients. While acknowledging these limitations, this study highlights the need for further research with larger and more diverse cohorts to validate and expand the findings. Such research could improve the reliability and applicability of the observed trends, thereby facilitating more informed clinical decision-making and improving patient outcomes in the management of pediatric pleural effusions.

## 5. Conclusions

Almost all pediatric PPE cases displayed biochemical characteristics typical of infectious pleural effusions. While PPE is generally characterized as an exudative effusion with a predominance of polymorphonuclear WBCs, this study revealed age-related differences: infants mainly showed mononuclear WBC predominance, while children exhibited polymorphonuclear WBC predominance, with a positive association between age and polymorphonuclear WBC percentage. Infants had higher pleural fluid glucose levels and lower total protein levels compared to children. Additionally, pleural fluid glucose levels were negatively associated with age, while total protein levels were positively associated.

These results underscore the importance of age-specific differences in interpreting and managing pediatric pleural effusions. By identifying distinct biomarker profiles for infants and older children, this study provides valuable insights that can guide clinical decision-making and promote age-appropriate strategies for managing PPE.

## Figures and Tables

**Table 1:** Diagnostic classification and treatment characteristics by age group through Spanish Pediatric Association guidelines [1] and demographic data of patients and descriptive statistics of pleural fluid biomarkers by age group in parapneumonic pleural effusion.

	Infants (*n* = 14)	Children (*n* = 40)	*p*	Treatment
**Diagnostic classification and treatment characteristics**				
Non-complicated PPE, *n* (%)	4 (28.6)	13 (32.5)		Antibiotics
Light Complicated PPE, *n* (%)	4 (28.6)	9 (22.5)		Antibiotics
Simple Complicated PPE, *n* (%)	5 (35.7)	14 (35.0)	0.698	Antibiotics + pleural drainage
Empyema, *n* (%)	1 (7.14)	4 (10.0)		Antibiotics + pleural drainage
**Demographic data**				
Age (years)	0.5 (0.0–1.0)	7.0 (2.0–11.0)	**<0.001**	
Sex (Male/Female), *n* (%)	4 (28.6)/10 (71.4)	16 (40.0)/24 (60.0)	0.446	
**Pleural fluid Biomarkers**				
RBCs (cells/µL)	2250.0 (675.25–18,000.0)	2800.0 (1000.0–8000.0)	0.860	
WBCs (cells/µL)	3455.5 (878.8–23,565.0)	2323.0 (490.0–6422.0)	0.333	
PN predominance (patients), *n* (%)	3 (21.4)	27 (67.5)	**0.003** ^†^	
Total Proteins (g/dL)	3.1 (2.4–3.6)	4.5 (3.9–4.8)	**0.001** ^†^	
Glucose (mg/dL)	92.5 (1.0–114.0)	32.5 (7.0–61.0)	**0.036** ^†^	
LDH (U/L)	1239.0 (345.8–3981.8)	1365.0 (896.0–3026.0)	0.762	
ADA (U/L)	12.8 (7.8–73.3)	51.8 (34.7–65.5)	0.156	
pH *	7.35 (7.10–7.40)	6.90 (6.80–7.10)	0.235	

Values are expressed as median (interquartile range) unless stated otherwise. RBCs: red blood cells; WBCs: white blood cells; PN: polymorphonuclear WBC; LDH: lactate dehydrogenase; ADA: adenosine deaminase. * pH was only measured in 7 infants and 20 children. Significant differences are highlighted in bold. ^†^ Significant differences remained significant after bootstrapping the models.

**Table 2:** Associations between age (years) and pleural fluid biomarkers.

	Rho	*p*
RBCs (cells/µL)	−0.067	0.827
WBCs (cells/µL)	−0.265	0.108
PN predominance (%)	**0.485**	**<0.001 ^†^**
Total Proteins (g/dL)	**0.609**	**<0.001 ^†^**
Glucose (mg/dL)	**−0.262**	**0.043 ^†^**
LDH (U/L)	−0.096	0.493
ADA (U/L)	0.048	0.823
pH *	−0.189	0.468

Values are expressed in Spearman’s correlation coefficient (rho). Abbreviations: RBCs: red blood cells; WBCs: white blood cells; PN: polymorphonuclear WBC; LDH: lactate dehydrogenase; ADA: adenosine deaminase. Significant associations are highlighted in bold. * pH was only measured in 7 infants and 20 children. ^†^ Significant associations remained significant after bootstrapping the models.

## Data Availability

The data could be shared by the corresponding author upon reasonable request.

## Abbreviations

The following abbreviations are used in this manuscript:

PPE	Parapneumonic Pleural Effusion
LDH	lactate dehydrogenase
ADA	adenosine deaminase
PRUH	Puerto Real University Hospital
VRUH	Virgen del Rocío University Hospital
IQR	interquartile range
WBC	white blood cell
